# Medial temporal lobe activation during encoding and retrieval of novel face-name pairs

**DOI:** 10.1002/hipo.20014

**Published:** 2004-04-15

**Authors:** C Brock Kirwan, Craig EL Stark

**Affiliations:** 1 Department of Psychological and Brain Sciences, Johns Hopkins UniversityBaltimore, Maryland; 2 Department of Neuroscience, Johns Hopkins UniversityBaltimore, Maryland

**Keywords:** fMRI, human, parahippocampal gyrus, hippocampus, associative memory

## Abstract

The human medial temporal lobe (MTL) is known to be involved in declarative memory, yet the exact contributions of the various MTL structures are not well understood. In particular, the data as to whether the hippocampal region is preferentially involved in the encoding and/or retrieval of associative memory have not allowed for a consensus concerning its specific role. To investigate the role of the hippocampal region and the nearby MTL cortical areas in encoding and retrieval of associative versus non-associative memories, we used functional magnetic resonance imaging (fMRI) to measure brain activity during learning and later recognition testing of novel face-name pairs. We show that there is greater activity for successful encoding of associative information than for non-associative information in the right hippocampal region, as well as in the left amygdala and right parahippocampal cortex. Activity for retrieval of associative information was greater than for non-associative information in the right hippocampal region also, as well as in the left perirhinal cortex, right entorhinal cortex, and right parahippocampal cortex. The implications of these data for a clear functional distinction between the hippocampal region and the MTL cortical structures are discussed. © 2004 Wiley-Liss, Inc.

## INTRODUCTION

Evidence from human neuropsychological cases has demonstrated that the medial temporal lobe (MTL) is essential for declarative memory (Scovilleand Milner, 1957; Milner et al., 1998). However, the contributions ofvarious structures (hippocampal region, perirhinal cortex, entorhinal cortex,and parahippocampal cortex) are unclear. Several lines of evidence, includinganimal and human electrophysiology (e.g., Eichenbaum et al., 1994; Cameron et al., 2001), lesion studies (Vargha-Khadem et al., 1997; Mayes etal., 2002), and human imaging studies (Eldridge et al., 2000; e.g., Henke etal., 1997, 1999; Sperling et al., 2001; Yonelinas et al., 2001; Zeineh et al., 2003), have been used to suggest a functional distinction between the hippocampalregion (including the CA fields, dentate gyrus, and subiculum)and the surrounding cortical areas, including the perirhinal, entorhinal, and parahippocampal cortices. Researchers have proposed several different functionaldistinctions for the subdivisions of the MTL. For example, Aggleton and Brown have proposed that thehippocampus is concerned with recollective, associational,spatial, and multi-item aspects of declarativememory (Aggleton and Brown, 1999; Brown and Aggleton,2001). In contrast, the perirhinal cortex (and potentiallyother adjacent cortical regions) is concerned withfamiliarity and single-item aspects of declarative memory.Related (although not identical) models share a common theme in suggesting that the hippocampus supportsconjunctive (O'Reilly and Rudy, 2000; e.g., Sutherlandand Rudy, 1989) or relational (e.g., Cohen et al., 1997)encoding and retrieval or that the hippocampus associatesdistinct events (Wallenstein et al., 1998) and items(Cohen and Eichenbaum, 1993; Eichenbaum et al., 1996; Rolls and Treves, 1998) in memory. Thus, while the processes attributed to the hippocampus (recollection and conjunctive or associative encoding and retrieval)and the surrounding cortical structures (familiarity andsingle item or non–associative encoding and retrieval) bythese models are not identical, they share an importanttheme in the division of labor between the hippocampusand the surrounding cortical structures related to the formationor use of associations.

However, an alternative view suggests that while there will be divisions of labor within the MTL, the available data do not support a clean functional distinction between the hippocampus and surrounding cortical structures according to associative vs. non-associative (or recollectionvs. familiarity-based) components of declarative memory (Reed and Squire, 1999; Zola and Squire, 2000;Broadbent et al., 2002; Stark et al., 2002; Stark and Squire, 2001a, 2003; Squire et al., 2004). For example,Stark and colleagues demonstrated that recognition memory for single items and relational information was equally impaired in amnesic patients with damage limited to the hippocampal region, even after the amnesic patients' performance was equated with that of controlsby repeated exposure to the study items or by degradationof controls' performance (Stark et al., 2002; Stark and Squire, 2003). Also, a recent animal lesion study (Buckley and Gaffan, 1998) has shown that animals with bilateral damage to the perirhinal cortex are impaired on botha configural or associative learning task as well as on anitem–item paired associate task, which depends more onmemory for single-items, and less on configural memory.Thus, while it is clear that the hippocampal region isimportant for associative, recollective, episodic, conjunctive, and relational memory, it is not clear that the adjacent cortical structures are not also important for these same forms of memory or that the hippocampal region is not important for single–item,familiarity–based, or non–relational forms of memory.

Data from electrophysiology have often been taken to support the associative hypothesis of hippocampal function. For example,it has been observed that hippocampal neurons fire maximally to conjunctions of features (Eichenbaum, 2000; Suzuki and Eichenbaum,2000; Brown and Aggleton, 2001). However, it has also been reported that neurons in the parahippocampal gyrus (entorhinal cortex) fire to conjunctions (Fried et al., 1997) and that neurons in the hippocampus also appear to display single-featurecodes (Wood et al., 1999). Taken together, these findings do notprovide unequivocal evidence for a clean functional dissociation within the MTL according to the associative aspects of memory, as both the hippocampus and the cortical areas of the MTL both takepart in representing conjunctions as well as single features.

Neuroimaging data have also been used to address this hypothesis. One approach has been to use the Remember-Know (Tulving, 1985) task and to contrast activity during the recollective “Remember” responses with activity during the more familiarity-based “Know” responses (e.g., Henson et al., 1999; Eldridge et al., 2000).For example, using event-related functional magnetic resonanceimaging (fMRI), Eldridge et al. (2000) reported that “Remember” judgments were accompanied by greater levels of activation in thehippocampus than “Know” judgments, correct rejections, and misses. These data are consistent with a greater role for the hippocampalregion in “Remember” responses. However, not only are there alternative explanations (e.g., that any more detail-rich retrieval would elicit greater activity, be it a recollective retrieval or not; see Discussion), but, most importantly for the present purposes, Eldridge et al. (2000) also observed greater activity for “Remember” than “Know” responses in the parahippocampal gyrus most likely parahippocampal cortex) as well as in the hippocampus. Likewise, in neuroimaging studies designed to test the associative hypothesis directly, activity related to associative encoding or retrieval has frequently been observed in both the hippocampal region and the parahippocampal cortex (Henke et al., 1997; Henke et al., 1999; Dobbins et al., 2002; Davachi et al., 2003; Düzel et al., 2003; Ranganath et al., 2004; see Discussion; Yonelinas et al., 2001). Finally, in a recent report by Henson et al. (2003), evidence of activity associated with familiarity was reported in multiple regions within the anterior portions of the MTL that appear to include the hippocampal region as well. Accordingly, while these data support a role for the medial temporal lobe in associative memory, they do not clearly differentiate between the hippocampus and parahippocampal gyrus in this regard.

The present study used event–related fMRI to investigate the role of MTL structures in encoding and later retrieval of face-name pairs in which either the face-name association is successfully encoded and retrieved or in which the individual components of the memory are successfully encoded and retrieved, but their association is not. Thus, we can address whether regional MTL activity during encoding or during retrieval is affected by the addition of this clearly associative component of the memory (this approach has clear parallels to the source + item vs. item-only approach employed by Davachi et al., 2003; see Discussion). During encoding, participants were scanned while viewing a series of face-name pairs that they were told to memorize for a later test. Participants were then scanned during a recognition task using face-name pairs that were in one of three conditions: (1) intact from the study episode (the same name paired with the same face as at time of study), (2) recombined from study (a previously studied name and a previously studied face that had not been studied together), or (3) novel face-name pairs (neither the name nor the face were present at study). Participants were asked to determine whether the pair was intact, recombined, or new and their responses to the various stimulus types were used to determine the type of memory formed for each stimulus. Specifically, intact pairs judged as intact were taken as evidence of successful encoding and retrieval of both components and the association between them, whereas intact pairs judged recombined were taken as evidence of successful encoding and retrieval of the two components (the face and the name) but unsuccessful encoding or retrieval of the association between them. Importantly, participants were instructed at test that they would never be shown one novel and one repeated component and to therefore respond “New” if they believed either component to be novel. By contrasting the successful associative trials with the single- item trials (unsuccessful associative, or “non-associative”) at study and at test, we observed significant activity in several MTL areas, including the left amygdala (study), the right hippocampal region (study and recognition test), the right entorhinal cortex (recognition test), the left perirhinal cortex (recognition test) and the right parahippocampal cortex (study and test). Thus, we found that associative and non-associative memory encoding and retrieval activatedMTLstructures including the hippocampal region as well as the adjacent cortical structures.

## MATERIALS AND METHODS

### Participants

Informed consent was obtained from thirteen healthy righthanded participants (7 female, age range 19–33, mean age=22.7, recruited by advertisement). Ethics and institutional review board approval was obtained from the appropriate committees.

### Materials

Materials consisted of 270 face-name pairs. Face images were taken from a series of portrait-style color pictures and were presented simultaneously with names on a black background using the Cogent 2000 toolbox (Wellcome Department of Imaging Neuroscience) for Matlab (The MathWorks). Names were drawn from the Social Security Administration listing of the 100 most common names for each decade of the last century and were matched to the approximate age of the person pictured. Only first names were used.

### Task

**Figure 1:**
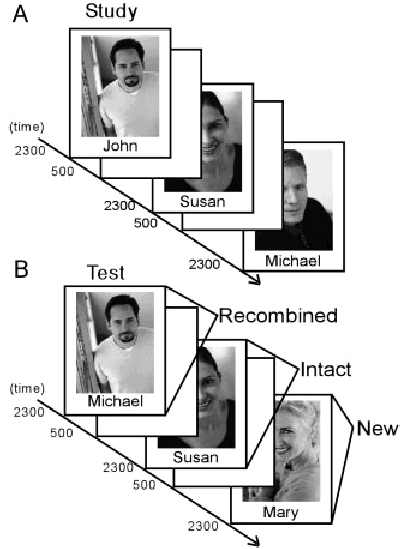
Behavioral design. A: Participants were scanned while studying novel face-name pairs. Pairs were presented for 2.3 s each with a 0.5-s ITI. B: Participants were also scanned during a recognition test in which face-name pairs were the same as at study (Intact face-name pairs), previously presented faces paired with different previously presented names (Recombined), or novel face-name pairs. At test, stimuli were again presented for 2.3 s with a 0.5-s ITI.

Participants were scanned during three study-test blocks. For the study phase, participants were told that they would be shown a series of 60 faces along with the pictured person's name and were encouraged to learn the people's names for a later memory test. Images were presented for 2.3 s with a 0.5-s inter-trial interval (see Fig. 1). During the test phase, participants were presented with 30 intact face-name pairs, 30 recombined faces and names, and 30 novel pairs. Recombined pairs consisted of faces and names that had both been presented at study, but in different pairs. Participants were explicitly told that the recombined pairs would have faces and names that had both been previously presented, but in different pairs, while the novel pairs would have new faces and new names. Thus, they were asked to indicate that the pair was novel if they felt that either member was novel. Participants were instructed to press one of three buttons with their right hand; the first if they thought the pair was intact from study, the second if they thought it was a recombined pair, and the third if they thought the pair was new. Stimulus presentation duration was the same for study and test phases. The entire study-test process was repeated three times for each participant using different stimuli each time.

Previous research (Stark and Squire, 2000; Stark and Squire, 2001a; Stark and Okado, 2003) has indicated that even while performing a retrieval task, there can be medial temporal lobe activity associated with incidental encoding of novel foils. To assess the degree of incidental encoding for each item during the recognition test, participants were given a surprise post-test outside the scanner after a delay of approximately 5 min (getting out of the scanner). Participants were shown the original 90 foil face-name pairs that were presented in the scanner along with 90 new facename foils and instructed to press one button on a computer keyboard marked “old” to items they had seen at any time in the scanner and another marked “new” to ones they did not recognize. Stimuli were presented at the same speed as in the scanner (2.3-s duration, 0.5-s ITI).

### fMRI Data Acquisition

Imaging was performed on a Philips 3T clinical MRI scanner equipped with an 8-channel sensitivity encoding (SENSE) head coil operating at a SENSE factor (R) of 3. By exploiting the sensitivity profiles of multiple surface coils, SENSE imaging can undersample k-space with fewer phase encoding steps, while still yielding full field of view (FOV) images that are free of aliasing. The result is significantly reduced acquisition time and distortion due to magnetic susceptibility (Pruessmann et al., 1999). Functional T2*- weighted images were acquired using an echoplanar, single shot pulse sequence with a matrix size of 80 × 80, TE of 30 ms, flip-angle of 90°, a TR of 1.4 s, and an in-plane resolution of 3 × 3 mm. For each scanning run, 28 oblique axial slices (3 mm thick with a 1-mm interslice gap) were acquired. Slices were aligned with the principle axis of the hippocampus, as determined by a series of sagittal localizer MRI images for each participant. Data acquisition began after the fourth image to allow for initial stabilization of the MRsignal. Following the three study-test blocks, a high-resolution (1 mm^3^) T1-weighted, 3-dimensional MP-RAGE sequence was acquired for anatomical localization (flip angle of 10°, field of view 256, acquisition matrix 256 × 256, 150 oblique axial slices).

### Image Analysis

Image analysis was performed using Analysis of Functional Neuroimages (Cox, 1996). Images were first co-registered through time using a three-dimensional registration algorithm. At this time, six vectors were created that code for all possible motion rotations and translations to be used later as regressors in a general linear model (GLM) of the fMRI data. Within each run, voxels were eliminated if the signal magnitude changed more than 30% between two samples or if the mean signal level was below a threshold defined by the noise inherent in the data. The three study blocks were concatenated, as were the three test blocks. Two GLMs were constructed: the first for the study and the second for the test data, using behavioral vectors coded according to stimulus type and participant response.

In analyzing the fMRI data from the study task, the behavioral data for study were coded and vectors created according to the stimulus type at test (Intact or Recombined) and the participant's response (Intact, Recombined, or New). Note that when stimuli were recombined at test, the elements of the study pair, the face and the name, were both used in two separate trials at test; effectively giving the participant two opportunities to demonstrate the degree of encoding for each of the recombined pairs.

Five vectors were created and entered into the GLM: Intact face-name pairs called Intact (II), Intact pairs called Recombined (IR), Recombined called Intact for both the face and the name (RI), and forgotten stimuli (Intact pairs called New or Recombined called New for both the face and the name). A vector for Recombined pairs called Recombined (RR) was not created so that this condition could serve as the baseline (see below). The remaining possibilities, when the participant correctly identified either the face or the name as either Intact or Recombined on one trial, but called the other New on the other trial, were combined into a single “Partial Single Item” (PSI) vector. This vector, while included in the GLM, was not analyzed any further. Table 1 shows the total number of trial types in each condition. In addition to these behavioral vectors, nine nuisance vectors were included in each GLM. Six vectors coded for head motion during the scan (three rotations and three transformations), two coded for drift in the MR signal (linear and second order) and one coded for trials in which the participant made no response.

**Table 1:** Mean Number of Raw Behavioral Responses and Percentages at Test

Stimulus response	Intact	Recombined	New
	Intact pairs		
Mean	57.9(10.2)	15.8(4.7)	11.8(8.7)
%	64.4(11.3)	17.6(5.3)	13.2(9.7)
	Recombined pairs		
Mean	23.8(10.5)	45.1(8.8)	15.2(8.4)
%	26.4(11.7)	50.1(9.7)	16.8(9.4)
	New pairs		
Mean	11.8(8.7)	15.2(8.4)	72.2(10.7)
%	13.2(9.7)	16.8(9.4)	80.2(11.8)

	All trials	New called new	
		
	No response	Remembered	Forgot

Mean	13.2(15.6)	48.1(13.8)	24.1(12.9)
%	4.9(5.8)	53.4(15.4)	26.8(14.3)

*Numbers are group mean number of responses (standard deviations shown in parentheses) and mean percentage of responses.

For participants to identify a face-name pair correctly as intact from study at the time of test, an associative memory for the face and the name must be formed, thus the II trials coded for associative memory. While it is possible to use an associative memory to correctly identify a recombined face-name pair as recombined (the RR trials), it is not necessary (this would be a “recall to reject” strategy). One need not remember the exact name or face associated with a given stimulus, only that this is not the correct pair. Therefore, as we cannot know how participants approach the RR trials, they cannot be used as a pure estimate of successful associative memory. As such, the II trials provide the purest estimate of successful associative memory. Similarly, in order for participants to identify intact pairs as recombined, they must have a single item memory for the face and the name, but they do not have a memory for the association between the two. Thus, the IR trials provide a clean example of non-associative memory. However, the converse, calling a recombined pair intact (RI trials), implies a false memory for an association that is not, in fact, there. Thus, while these trials were coded in the GLM, they were not considered an example of either associative or non-associative memory.

Test data were similarly coded according to stimulus type and participant response (see Table 1). The vectors included Intact pairs called Intact (II), Intact pairs called Recombined (IR), Recombined pairs called Intact (RI), Intact pairs called New (IN), Recombined pairs called New (RN), New pairs called New that were subsequently remembered at the post-test (NNR), and New pairs called New that were subsequently forgotten (NNF). Items that were New and called either Intact or Recombined were coded as false alarms (FA). Again, the II and IR items coded for associative and non-associative memory, respectively.

TheGLMwas constructed using a deconvolution technique (D. Ward, “Deconvolution Analysis of FMRI Time Series Data,” http://afni.nimh.nih.gov/afni), which first estimated the impulse response function within each voxel based on 11 time points (0–15.4 s) and then performed a multiple linear regression. The sum of the beta coefficients for the time points corresponding to the expected peak in the hemodynamic response was taken as the model's estimate of the response to each trial type (2.8–12.6 s). At both study and test, the RR items served as the baseline condition. The choice of the RR condition was somewhat arbitrary. Its use as a baseline is not meant in any way to represent an estimate of “zero” activity in the MTL (Stark and Squire, 2001b), for it is surely an active condition. All contrasts presented here were direct, bidirectional comparisons (mixed effects model with participants as a random effect and condition as a fixed effect) between activity in other conditions. As such, the contrasts factor out activity associated with whatever baseline was used, making the choice of baseline almost irrelevant (for example, a separate parallel analysis of the fMRI data with a different baseline, the New pairs called New and subsequently remembered, should not and did not produce different results). Thus, as the RR trials were not of critical interest, and as there were a consistently large number, they were selected to serve as the “baseline” in the GLM.

Initial spatial normalization was accomplished using each participant's structural MRI scan to transform the data to the atlas of Talairach and Tournoux (1988). In this process, statistical maps were resampled to 2.5 mm^3^. Statistical data from all subjects were then smoothed using a Gaussian filter with a full-width half-maximum of 4 mm to help account for variations in the functional anatomy. In order to achieve better alignment of the medial temporal lobe, the Region of Interest Alignment technique (ROI-AL) was used (Stark and Okado, 2003). The technique begins by defining the structures in the MTL (hippocampal region, temporal polar cortex, perirhinal cortex, entorhinal cortex, and parahippocampal cortex) bilaterally according to the techniques described by Insausti et al. (1998). Insausti et al. differentiate the temporal polar cortex from the rest of the perirhinal cortex. However, as there is a lack of consensus as to whether there is a functional dissociation between these regions in the human literature, we refer to this area as the temporal polar-perirhinal cortex when reporting our results. The parahippocampal cortex was further defined bilat- erally as the portion of the parahippocampal gyrus caudal to the perirhinal cortex and rostral to the splenium of the corpus callosum (this definition of the rostral extent includes only slightly more tissue than that defined by Preuessner et al., 2002) The hippocampal region (the CA fields of the hippocampus, the dentate gyrus, and the subiculum) was also defined bilaterally. ROI-AL uses these anatomically defined ROIs to calculate an additional 12-parameter transformation matrix that attempts to fine-tune the cross-participant alignment in the MTL by maximizing the overlap of anatomically defined ROIs (here, participants' ROIs within the MTL were aligned to a model of the MTL generated using a bootstrap technique that iteratively aligned 20 other participant's ROIs and calculated a modal ROI label for each voxel). In addition to improving cross-participant alignment, the ROI-AL technique can localize results from cross-participant analyses to specific anatomically defined regions of interest (e.g., perirhinal vs. parahippocampal cortices) by comparing the location of each active region to a composite anatomical model based on the mode of the individual subjects' anatomically defined regions of interest. Unfortunately, coordinates in a standardized reference frame (e.g., Talairach or MNI coordinates) are distorted somewhat in this process and data well outside the aligned regions can be grossly distorted. Thus, the coordinates reported in the present study should be only be taken as approximations of Talairach space.

To examine the key contrast between associative (II) and nonassociative (IR) trials, a voxel-wise, bidirectional, two-tailed paired *t*-test was conducted between the II and IR conditions (such that participants are treated as a random effect and condition is treated as a fixed effect). This contrast was used to identify any functional ROIs that passed a statistically significant voxel-wise threshold (*P* < 0.03) and a spatial extent threshold of 219 mm^3^ (*P* = 0.053, correcting for multiple comparisons). In addition, contrasts were performed at study between II and Forgotten trials, IR and Forgotten trials, and at test between NNR and NNF trials to examine subsequent memory effects using the same statistical thresholds.

## RESULTS

Overall, at test participants were 64.9% correct on the threealternative forced-choice recognition memory test (range 51.1–75.2%). The mean II response rate was 64.4%. The mean RR response rate was 50.1%. The mean false alarm (FA) rate was 16.7% (identifying Intact or Recombined pairs as New), while the mean rate for calling Recombined pairs Intact was 26.4%. The mean correct rejection rate for the New pairs was 80.2% (NNR and NNF conditions). For the conditions in the critical contrast, the II and the IR conditions, the mean number of responses was 57.9 and 15.8, respectively (see Table 1). Collapsed across stimulus type, there was a slight response bias. The average total number of “New” responses was 99.2, “Recombined” responses averaged 73.3, and “Intact” averaged 84.3. Pairwise *t*-tests revealed that the difference between the number of New responses differed significantly from Recombined (t(12) = 3.83, P < 0.01), while the difference between the number of New vs. Intact and Recombined vs. Intact was not significant (t(12) = 1.61, *P* = 0.13 and t(12) = 2.00, *P* = 0.07, respectively). Participants were more likely to categorize old stimuli (those that had been presented at study, collapsed across Intact and Recombined pairs) as Intact (mean responses = 81.7) than Recombined (mean = 60.9) or New (mean = 27.0). *t*-tests revealed that these differences were significant: t(12) = 3.71, *P* < 0.01, t(12) = 7.04, *P* < 0.001, and t(12) = 5.52, *P* < 0.001 for the difference between Intact vs. Recombined, Intact vs. New, and Recombined vs. New, respectively.

Although an associative strategy would be the most effective way to solve this task, it is worth considering the possibility that participants based their responses to previously encountered stimuli on the relative item familiarity of those stimuli and treated the three response options as three points on a confidence-rating scale. Thus, collapsed across Intact and Repaired stimulus types, stimuli that were well encoded would elicit an “Intact” response, while stimuli that were less well-encoded would elicit a “Recombined” response, and stimuli that were not encoded would be called “New.” While this might explain behavioral phenomena such as the high number of RI responses (26.4%), it does not entirely explain the behavioral data. Participants performed significantly above chance for both the recombined (t(12) = 6.20, *P* < 0.0001) and intact stimuli (t(12) = 9.87, *P* < 0.0001), indicating that they did in fact correctly perform the task using an associative strategy. Thus, the relative memory strength of the II versus the IR conditions cannot be driving the observed behavior.

It should be noted that while a large average number of trials were included in the II condition (57.9), there were far fewer in the IR condition (15.8; see Table 1). While additional trials in the IR condition would certainly decrease the noise in our estimate of the amplitude of IR responses, the overall mean amplitude of the activation will not change with the smaller numbers. Thus, the increase in the amount of noise or variation around this mean resulting from this decreased sample size could serve to reduce our ability to resolve II vs. IR effects.

### Subsequent Memory Analysis

**Figure 2:**
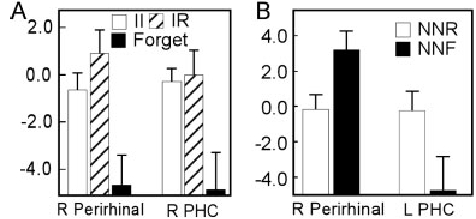
Predictive memory effects at time of study (A) and at time of test (B). At study, II (white) and IR (hashed) trials were combined and their activity was contrasted with the activity associated with subsequently forgotten trials (black). Two MTL regions, one within right perirhinal cortex and one within right parahippocampal cortex, exhibited an overall predictive memory effect. At test, activity associated with new items remembered outside the scanner (NNR; white) was contrasted with activity associated with new items forgotten outside the scanner (NNR; black). Two MTL regions, one within right perirhinal cortex and one within left parahippocampal cortex showed predictive memory effects. The vertical axis represents activity (sum of beta coefficients) and error bars indicate the standard error of the mean.

Several studies have demonstrated predictive memory effects in the MTL, where activity for remembered items is greater than activity for subsequently forgotten items (Brewer et al., 1998; Wagner et al., 1998; Fernandez et al., 1999, 2002; Otten et al., 2001; Davachi and Wagner, 2002; Strange et al., 2002; Davachi et al., 2003; Stark and Okado, 2003). To examine overall subsequent memory effects, unbiased with respect to associativity, a voxel-wise analyses of the fMRI data from the study phase was conducted which contrasted activity during encoding of subsequently remembered items (collapsed across II and IR conditions) with activity for subsequently forgotten items (Forgot condition). Significant Remembered vs. Forgotten predictive memory effects were observed in regions within the right perirhinal cortex (Talairach coordinates of approximate ROI center = 26, 8, −22), and within the right parahippocampal cortex (25, −29, −22). In both areas, activity associated with subsequently remembered items was greater than forgotten. Within both areas, activity for II and IR conditions was similar (perirhinal ROI t(12)=−1.57, *P* = 0.14; parahippocam- pal ROI t(12) = -0.24, *P* = 0.82) (Fig. 2A). Averaging across II and IR conditions may have obscured predictive memory effects for just associative or non-associative information. To rule out this possibility, further voxel-wise *t*-tests were conducted on the II vs. Forgotten and IR vs. Forgotten contrasts. The II vs. Forgotten contrast revealed a significant difference in an almost identical region of the right parahippocampal cortex as was revealed in the overall Remembered vs. Forgotten contrast (24 overlapping voxels). The IR vs. Forgotten contrast revealed a significant difference in an overlapping area (17 overlapping voxels) in the right perirhinal cortex. In neither the II vs. Forgotten, nor the IR vs. Forgotten contrast was a new MTL region revealed that might suggest a special role in associative or single-item memory encoding that could have been obscured by the overall Remembered vs. Forgotten contrast. Activity in the perirhinal and parahippocampal cortices predicted subsequent memory regardless of associative condition.

To examine the predictive memory effect at the time of test for novel items (Fig. 2B), we contrasted activity for new items correctly called new that were subsequently remembered at the posttest with those that were subsequently forgotten (NNR vs. NNF). Significant differences were found in regions within the right perirhinal cortex (26, 0,−31) and within the left parahippocampal cortex (−30, −40, −9). Consistent with the report of (Stark and Okado, 2003), activity for remembered items was greater than for forgotten items in the left parahippocampal cortex. Activity predictive of subsequent memory of the foil items was also observed in the right perirhinal cortex, but of interest, the subsequently remembered items were associated with less activity than subsequently forgotten items here.

The finding of greater activity for remembered items (regardless of associative condition) relative to forgotten items in the parahippocampal and perirhinal cortices is consistent with much of the literature on subsequent memory effects (Brewer et al., 1998; Wagner et al., 1998; Fernandez et al., 1999; Otten et al., 2001; Davachi and Wagner, 2002; Fernandez et al., 2002; Strange et al., 2002; Davachi et al., 2003; Stark and Okado, 2003). However, the pattern of results observed in the right perirhinal cortex during retrieval is apparently inconsistent with this. While it is showing a predictive effect, the direction is opposite to the traditional pattern. It is difficult to draw any strong conclusions about the nature of this reversal from the data at hand. We would note first that the two ROIs showing subsequent memory effects within the perirhinal cortex were not overlapping: the ROI from the study condition was more anterior to the ROI from the test condition. In the study condition, participants were explicitly instructed to memorize the stimuli for a later memory test, while during the test condition, the stimuli in the subsequent memory analysis were only incidentally encoded, as the participants were never told that they would be tested on the novel foils. The only report of subsequent memory effects within the MTL for items encoded during a retrieval task only reported activity following the traditional pattern (Stark and Okado, 2003). However, the retrieval task used here and in Stark and Okado (2003) significantly differ. We would also note that during retrieval, old vs. new contrasts have been associated with both positive-going and negative-going responses, the latter associated with both repetition priming effects (Schacter and Wagner, 1999) and familiarity signals (Henson et al., 2003). From the present data, however, the source of this reversal of effect cannot be determined but does suggest future study.

### Associative vs. Non-associative

**Figure 3:**
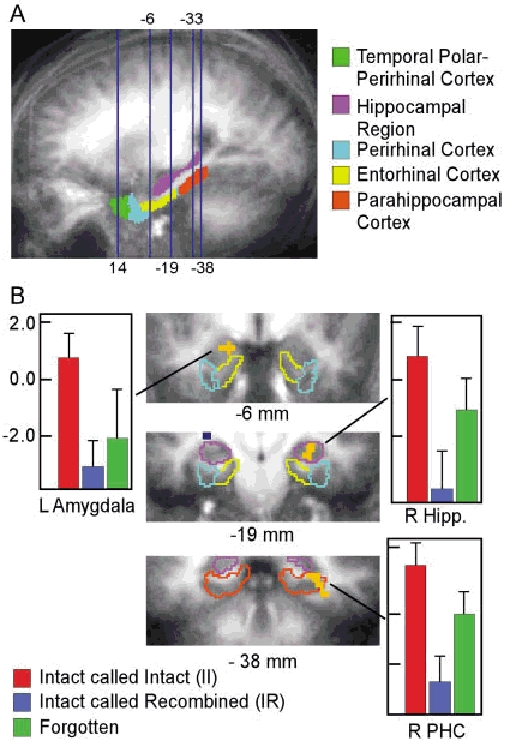
Locations of slices shown and fMRI data from the Study phase. A: Sagittal section (averaged across participants after ROI-AL alignment) showing the approximate locations (blue lines) of coronal slices presented here and in Fig. 3. Locations of the anatomically defined regions in one hemisphere of the model used for ROI-AL alignment and localization of results (hippocampal region, temporal polar cortex, perirhinal cortex, entorhinal cortex, and parahippocampal cortex) are shown as color overlays. B: fMRI data from the Study phase are shown in cropped coronal slices (left side of image is the left side of the brain; average across participants; approximate slice location indicated below image) indicating activity in MTL regions (orange overlay; regions outside the MTL are coded in blue) that differed between the pairs that were subsequently Intact called Intact (II) and pairs that were subsequently Intact called Recombined (IR). Colored outlines show the location and extent of the anatomically defined regions within the MTL with colors corresponding to those in (A). Bar graphs show the activity (sum of beta coefficients) within each functionally defined ROI associated with II (red), IR (blue), and subsequently forgotten trials (green). On each bar, the standard error of the mean across participants is indicated.

In the direct, bidirectional contrast to assess associative vs. nonassociative activity during the study phase, we observed significant differences in activity for face-name pairs in which the association between the face and name was later remembered (II) versus facename pairs in which the components were remembered without the association (IR) in three regions within the MTL (Fig. 3). These functionally defined ROIs were located in a region within the right hippocampal region (31, −23, −8), a region within the right parahippocampal cortex (32, −39, −4), and a region within the left amygdala (−20, −5, −11) (the amygdala is close enough to the structures of the MTL defined in the ROI-AL technique to be well aligned and thereby allow us to identify any activity as being a distinct locus within the amygdala with some confidence). Thus, activity predicting whether the associational component of a facename pair would be remembered or whether it would be forgotten and only the individual components would be remembered was observed both in the hippocampal region and the parahippocampal cortex.

**Figure 4:**
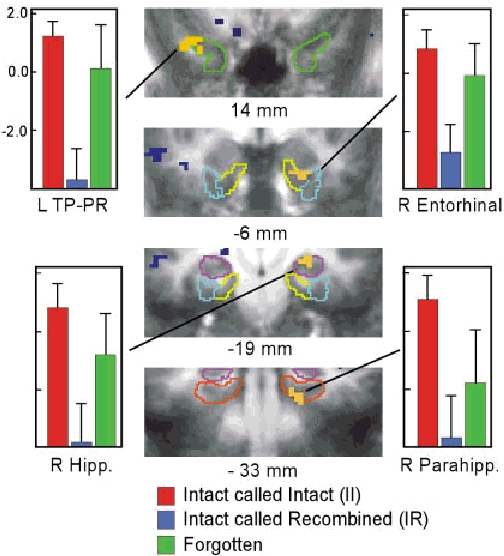
fMRI data from the Test phase are shown in cropped coronal slices (left side of image is the left side of the brain; approximate slice location indicated below image) indicating activity in MTL regions (orange overlay; regions outside the MTL are coded in blue) that differed between Intact called Intact (II) and Intact called Recombined (IR) trials. Colored outlines show the location and extent of the anatomically defined regions within the MTL with colors corresponding to those in (Fig. 3A). Bar graphs show the activity (sum of beta coefficients) within each functionally defined ROI associated for II (red), IR (blue), and Forgot (average of IN and RN; green) trials. On each bar, the standard error of the mean across participants is indicated.

At the time of the test (Fig. 4), significant differences in MTL activity for associative vs. non-associative memory (II vs. IR) were observed in regions within the left temporal polar-perirhinal cortex (−41, 14, −18), the right entorhinal cortex (22, −7, −24), the right hippocampal region (24, −16, −7), and the right parahippocampal cortex (19,−33,−14). Thus, like activity during study, both the hippocampal region and structures in the adjacent cortex of the MTL were more active during associative than non-associative memory retrieval.

To investigate the role of the specific areas identified as involved in successful encoding at time of retrieval, we used the same ROIs defined at the time of encoding to investigate the data from retrieval. Paired *t*-tests revealed a significant difference between the II and IR condition in the right hippocampal ROI (t(12) = 2.23, *P*<0.05). However, the converse analysis, investigating the ROIs from retrieval at time of encoding, did not show a significant difference between the II and IR conditions in the right hippocampal ROI. Although the two ROIs in the right hippocampus overlap by 4 voxels, the ROI from the encoding condition is more anterior than that from retrieval. No other significant differences were detected in any of the other ROIs in either analysis.

Although the hippocampal region and the adjacent cortical structures of the MTL both showed greater activity for associative relative to non-associative memory during encoding and retrieval, it is still possible that the hippocampal region might show a larger associative vs. non-associative effect than the adjacent cortical structures. To assess whether the hippocampal region might be more active in the associative component of declarative memory relative to the structures in the parahippocampal gyrus, the magnitude of the associative vs. non-associative effect (II vs. IR) was compared across regions. Any increase in the difference between activity in these two conditions across regions could be taken as evidence supporting a differential role in the associative component of declarative memory. (Alternatively, greater activity associated with II than IR trials in general could simply be the result of stronger or more robust memory for II than IR trials.) Analysis of variance (ANOVA) contrasting activity in the II and IR conditions across ROIs revealed no significant interaction between condition and ROI at study (F(2,24)=0.24, *P*=0.78) or at test (F(3,36)= 0.69, *P* = 0.57). Therefore, our study did not find evidence for specialization of function within the MTL according to a distinction between associative versus non-associative memory.

One possible explanation of the observed activity (II > IR) is that the relative item memory in the II condition is greater than that of the IR condition. In the II condition, participants must have item memory for both the face and the name as well as a memory for the association between the two. However, in the IR condition, participants lack the memory for the association. This lack of memory may be due to poorer overall encoding of the stimuli in the IR condition at the time of study. Thus, it is possible that the individual components of the face-name pair were themselves more poorly encoded in the IR condition, and the II and IR conditions differ in the level of single item memory associated with each. This reasoning would of course extend to stimuli that were Forgotten, which would have shown the poorest encoding, and thus show even less overall activity than either II or IR conditions. However, when we examined activity within the functionally defined ROIs for the Forgotten condition, we found activity that was in between the levels of the II and IR conditions in each of the ROIs at study (green bars in Fig. 3; see Davachi et al., 2003, for similar results). These differences were statistically reliable in the right hippocampal region (II > Forgot, t(12) = 2.69, *P* < 0.05; Forgot > IR, t(12) = −2.17, *P*= 0.051) and in the right parahippocampal cortex (II > Forgot, t(12) = 2.64, *P* < 0.05; Forgot > IR, t(12) = −2.45, *P* < 0.05). A similar pattern was observed in the fMRI data from the test phase (Fig. 4). Within the ROIs functionally defined by the II vs. IR contrast, activity for forgotten items (green bars in Fig. 4) was less than the II condition, but greater than for the IR condition. The difference between the forgotten and IR conditions was statistically reliable in the left temporal polar-perirhinal cortex (t(12)= −2.50, *P* < 0.05), the right entorhinal cortex (t(12)=−3.47, *P*<0.01), and in the right hippocampal region (t(12) = −2.31, *P* < 0.05). Thus, in the ROIs selected to show differences in activity between associative (II) and non-associative (IR) trials, activity associated with subsequently forgotten trials was in-between the other two indicating that the observed difference in activity is due to something other than the relative item memory strength in each condition. We should note that in these analyses, the selection of voxels by the II vs. IR contrast does not bias the activity for the Forgotten items to take on any value and they, along with all the conditions except the RR baseline condition (defined as zero), are free to vary in the deconvolution analysis. Thus, an interpretation that the Forgotten activity represents a “regression to the mean” is not a valid account of the data (e.g., many of these same voxels would have been selected in a II vs. Forgotten contrast and would exhibit IR activity below both rather than at the mean of both). It is worth noting that when an ANOVA (II vs. IR vs. Forgotten) is used to functionally define the ROIs, similar results are obtained, albeit with less statistical power than with the direct II vs. IR *t*-test.

## DISCUSSION

The present study examined the role of various MTL structures in the encoding and retrieval of associative versus non-associative memories. Participants were scanned during learning and later recognition testing of a series of novel face-name pairs. Based on the stimulus type and the participants' responses, we were able to determine if participants had formed an associative link between the face and the name or whether they had formed a memory for the individual components, but not for the association between the two. There was greater activity at time of encoding for associative than for non-associative information in several MTL structures: the right hippocampus, right parahippocampal cortex, and the left amygdala. A similar pattern of activity was found during retrieval, with greater activity for associative than for non-associative trials in left temporal polar-perirhinal cortex, right entorhinal cortex, the right hippocampal region, and the right parahippocampal cortex. Particularly telling against the hypothesis that the hippocampus alone supports associative memory while the adjacent cortical areas support non-associative memory is the fact that there was not a proportional difference in the activity for associative versus nonassociative memory. The difference between activity for associative and non-associative memory in the hippocampus was not greater than the difference in any of the other areas reported here.

It should be noted that a significant amount of the data from animal studies are focused on a distinction between the hippocampus and the perirhinal cortex in particular (for review, see Brown and Aggleton, 2001). The present study speaks to that specific distinction in so far as the activation found at time of retrieval in the temporal polar-perirhinal cortex can be considered as truly being within the perirhinal cortex. Unfortunately, there is not a consensus as to the exact boundaries of perirhinal cortex in the human and whether the more anterior temporal polar region should be considered a functionally distinct region from the more posterior portion of perirhinal cortex. That said, the present data clearly address the common and more general dissociation that is often made between the hippocampus and the adjacent cortical structures that lie along the parahippocampal gyrus (the entorhinal, perirhinal, and parahippocampal cortices).

While these results may seem to be in contrast to the existing neuroimaging literature that has been used to support the view that the hippocampal region is specifically involved in the recollective or associative component of declarative memory, we would suggest that the present results are largely consistent with these existing data. Here, we attempt to review the relevant studies to show that many (although not all) of the present results can be found in similar forms in other neuroimaging studies in the literature and that, when viewed as a whole, the existing literature do not support a clear functional dissociation between the hippocampal region and the adjacent cortex according to recollective or associative versus familiarity or single-item based memory.

### Studies of Recollection and Familiarity

While the present study assessed activity correlated with associative aspects of declarative memory, several others have used the Remember/Know paradigm (Tulving, 1985) to investigate the role of MTL structures in judgments of recollection and familiarity. To the extent that “Remember” or recollective responses approximate or are at least correlated with the associative (II) condition of the current study and “Know” responses approximate the non-associative IR condition, the findings from the Remember/Know task can be related to those of the present study. In one study, Henson et al. (1999) reported little difference within the MTL between “Remember” and “Know” conditions at test and only a small region in the parahippocampal gyrus showing less activity at time of study for subsequent “Remember” responses than “Know” responses. In contrast to this mostly null result, Eldridge et al. (2000) reported greater activity for “Remember” than “Know” judgments, correct rejections, and misses in several regions including the hippocampal region itself. These data are consistent with a greater role of the hippocampus in recollection than familiarity judgments. However, there are some limitations to the Remember-Know task that complicate this interpretation. First, this task is difficult to perform in the fMRI scanner since it requires two steps to avoid becoming a simple confidence rating (Hicks and Marsh, 1999). Second, it is difficult to entirely ascribe the enhanced activity for “Remember” responses to a functional dissociation favoring the recollective component of recognition. It is quite plausible that any detail-rich retrieval would result in more activity than a detail-poor retrieval. As such, “Remember” responses might yield more activity than “Know” responses in a region not particularly involved in the recollective component itself. Finally, even if one reasonably assumes that some portion of the enhanced activity for “Remember” responses over “Know” responses can be attributed to recollective or associative aspects of processing, Eldridge et al. (2000) observed greater activity for “Remember” than “Know” responses not only in the hippocampal region, but also in the posterior parahippocampal gyrus as well. It is worth noting that activity associated with correct rejections showed a different pattern in the two regions. In the hippocampal region, “Know” and correct rejection responses were similar, whereas in the posterior parahippocampal gyrus, “Know” responses were associated with greater activity than correct rejections. This difference in the activity for correct rejections could indicate a dissociation according to recollection vs. familiarity or it could result from activity associated with incidental encoding of the novel foils (for further discussion, see Stark and Squire, 2003).

In addition to recollective activity outside of the hippocampal region, there is some evidence for familiarity-related activity in the hippocampal region itself. Henson et al. (2003) report activity associated with familiarity in several studies within anterior portions of the MTL that appear to include entorhinal/perirhinal cortices as well as the hippocampal region. Similarly, Stark and Squire (2000, 2001a) report activity in the hippocampal region during simple recognition memory tasks that can rely purely on familiarity, with no apparent increase in activity when the task becomes more associative or recollective (Stark and Squire, 2001a). Thus, the neuroimaging data using measurements of recollection and familiarity do not support a clean functional dissociation between recollective processing in the hippocampal region and familiarity processing in the structures of the parahippocampal gyrus.

### Associative Memory Studies

A number of recent neuroimaging studies have directly examined associative memory formation and retrieval directly within the MTL. Many of these studies report greater activity for associative than non-associative memory within the hippocampus. For example, Sperling and colleagues (Sperling et al., 2001) reported significant encoding-related activity for face-name pairs within the hippocampus (defined as the hippocampus proper, subiculum, and entorhinal cortex) but not elsewhere in the parahippocampal gyrus. Here, the authors reported greater activity during the viewing of blocks of novel face-name pairs with that while viewing blocks of repeated face-name pairs. However, the blocked design of this study did not allow isolation of individual encoding trials based on the quality or amount of information subsequently retrieved. Thus, it is difficult to say if the observed activity is due to encoding of the individual components of the face-name pair (the faces and the names), encoding the association between the pair components, or due to novelty detection (Strange et al., 1999). In a subsequent study using an event related fMRI design, Sperling et al. (2003) reported large activations in the hippocampus and parahippocampal gyrus for successful associative encoding relative to unsuccessful (failed) encoding. Small et al. (2001) restricted their analysis to the hippocampal region and report activity for the encoding and retrieval of individual faces and names as well as for encoding and retrieval of face-name pairs. Notably, the activity for face-name associations was more widespread than the combination of activity for faces and for names alone. However, by restricting the analysis to the hippocampal region, it is unknown whether this enhanced activity for the face-name associations would have been observed elsewhere.

However, other studies of associative memory in the MTL have shown increased activity for associative relative to non-associative memory in the hippocampal region as well as in the parahippocampal gyrus. For example, in a recent study, Jackson and Schacter (2004) scanned participants while performing a verbal associative encoding task. Task trials were later binned according to performance on a post-scan associative recognition test, much the same as in the present study. The authors report greater activation for successful associative binding (analogous to the II condition in the present study) relative to unsuccessful associative binding (analogous to the present IR condition) in the MTL in bilateral entorhinal/ perirhinal cortex as well as in left hippocampus. Henke and colleagues used positron emission tomography (PET) to examine activity during encoding and retrieval of face-house pairs in one study (Henke et al., 1997) and fMRI to examine encoding of semantic associations between words (Henke et al., 1999). In both studies, associative activity was greater than non-associative in the hippocampal region and in the parahippocampal gyrus. In a study of recognition memory, Yonelinas and his colleagues (Yonelinas et al., 2001) reported activity greater for associative than non-associative in the hippocampus and the posterior parahippocampal gyrus. A recent study by Zeineh et al. (2003) that was able to localize activity to the specific subregions of the hippocampal region showed increased activity in the hippocampal region (localized to CA2, CA3, and dentate gyrus) and in the parahippocampal cortex during encoding (and parahippocampal cortex activity during recall). Furthermore, Pihlajamaki et al. (2003) observed activity during encoding of picture pairs in the hippocampus, but found this activity more consistently in the perirhinal cortex. Killgore et al. (2000) report greater activity for associative than non-associative encoding in the amygdala as well as in the hippocampus. Since this study restricted analysis to these two areas, it is unknown whether associative activity occurred in the parahippocampal gyrus.

Consistent with many of the above studies, the present study found greater activity for associative than non-associative encoding and retrieval in the hippocampal region as well as in areas of the parahippocampal gyrus. Areas showing this pattern of activation during encoding include the left amygdala, right hippocampus, and right parahippocampal cortex. At time of retrieval, the right hippocampus, right parahippocampal cortex, right entorhinal cortex, and left perirhinal cortex also showed this same pattern of activation. Interestingly, Düzel et al. (2003) showed a similar pattern of activity in the parahippocampal-perirhinal cortex with greater activity during a recognition test for familiar relative to novel associative configurations. The hippocampus showed the opposite pattern of results, with activity for novel configurations greater than for familiar ones. In both conditions, the relative item familiarity of the items in the configurations was the same, while the configurations of the items was manipulated.

Davachi and colleagues have reported data suggesting a functional distinction within the MTL (Davachi and Wagner, 2002; Davachi et al., 2003). In a compelling study investigating item versus item plus source memory (Davachi et al., 2003), an area in the left perirhinal cortex predicted overall subsequent memory that was not sensitive to whether the source was subsequently remembered or not. The MTL activity that predicted subsequent source retrieval was more wide-spread, however, with activations in the bilateral hippocampal region and left parahippocampal cortex. The authors interpreted these data as indicating that perirhinal cortex was associated with encoding of the item itself, whereas the hippocampus (and parahippocampal cortex) was associated with encoding of the source information. The present study replicates these findings insofar as we find activity in the perirhinal cortex (on the right in the present study) that predicts subsequent memory irrespective of the associative memory condition and activity in the hippocampal region and the parahippocampal cortex that predicts subsequent successful associative memory. Notably, however, we also found the non-associative pattern of encoding-related activity in a separate region of the parahippocampal cortex (the two areas of activation in the parahippocampal cortex in the present study do not overlap with each other.)

Thus, the data from the study phase replicate the findings of Davachi et al., (2003) but include the observation of an overall, non-associative subsequent memory pattern of results in a separate area of the right parahippocampal cortex and an observation of the associative subsequent memory effect in the left amygdala. In addition, here, we present data from a retrieval task where activity related to the successful retrieval of the association (relative to retrieval of the items only) was observed in the left perirhinal cortex, the right entorhinal cortex, right hippocampus, and right parahippocampal cortex. Thus, while we clearly replicate many of the findings reported by Davachi et al. (2003), the conclusions with respect to the division of labor within the MTL are not as clear. Perirhinal cortex appears non-associative at study, but associative at test (see Dobbins et al., 2002, for another report of perirhinal activity being a combination of associative and non-associative information). Parahippocampal cortex appears to be both associative and non-associative at study, depending on where in parahippocampal cortex one looks. Overall, at test the clear pattern is to exhibit greater activity for associative than non-associative memory throughout the collection of structures in the MTL. Thus, it is difficult to use the present data to support a basic dichotomy between an associative hippocampal region and a non-associative perirhinal cortex or parahippocampal gyrus.

### Forgotten Items

One other aspect of our results that mirrors those of Davachi et al. (2003) warrants discussion. In both studies, associative encoding activity was found to be greater than activity for items subsequently forgotten, which was in turn greater than activity for nonassociative encoding. While Davachi et al. (2003) did not report data from a retrieval task, the present study demonstrates that this pattern of activity can also be present at retrieval (Fig. 4). One possible explanation of this pattern of activity (associative > forgotten >non-associative) at the time of retrieval is that the forgotten items were weakly encoded during the study phase. Thus, at time of test, the participant judges these items as new, and the observed activity is the result of incidental encoding of these “novel” items at the time of test. This explanation does fit with other data regarding automatic encoding of foils during a recognition memory paradigm (Stark and Okado, 2003; Stark and Squire, 2000, 2001a), as well as with other subsequent memory effects observed in the present study. Incidental encoding at time of test, however, is probably not the most complete explanation of the data, as the same pattern of results was observed at study, both here and in Davachi et al. (2003).

Another possible explanation for this pattern (associative >forgotten > non-associative) can be found by turning to the hypothesis that associative recollection and non-associative familiarity are mediated by separate neural mechanisms and structures (for review, see Brown and Aggleton, 2001). If a structure were involved in the encoding and retrieval of an associative component, it would certainly exhibit greater activity in our associative II condition than our non-associative IR condition. Exhibiting greater activity for associative items than for forgotten items, which in turn show greater activity than for non-associative items at time of study may imply that an associative memory can be created (and is mediated by this structure) in the absence of single-item or non-associative memory for the components. At time of test, this hypothesis would make a similar prediction with forgotten activity being elevated as a result of incidentally encoding an association that cannot later drive recognition performance (the re-encoding of these Forgotten items). In contrast to such an “associative structure”, a “non-associative structure” might exhibit similar levels of activity for our associative II condition and our non-associative IR condition, as both conditions must include the non-associative component. Davachi et al. (2003) used this line of reasoning to support a division of labor within the MTL, as the hippocampal region and parahippocampal cortex could be viewed exhibiting the pattern of an associative structure and the perirhinal cortex could be viewed as exhibiting the pattern of a non-associative structure. However, even if we interpret the associative > forgotten > non-associative pattern of activity as representing successful encoding or retrieval of the associative component we are left with the conclusion that the amygdala, the perirhinal cortex, entorhinal cortex, parahippocampal cortex and the hippocampal region can all show this pattern of activity indicative of an “associative structure.”

## CONCLUSIONS

The current results indicate that activity correlated with associative encoding and retrieval is greater than activity correlated with non-associative encoding and retrieval throughout the MTL, including not just the hippocampal region, but also the adjacent cortical structures. Accordingly, while the present data and the data just reviewed cannot be resolute with respect to function (all are correlational datasets), they indicate that the hippocampal region may be extending the processing done by nearby MTL cortical regions, but they do not support a simple functional division within the medial temporal lobe according to associative versus non-associative components of memory. That activity in the hippocampal region is correlated with associative, recollective, or source components of declarative memory is quite clear. However, it is equally clear that activity in the adjacent cortical structures (most notably the parahippocampal cortex) is also correlated with these forms of memory as well. Thus, we suggest as have others (e.g., Lavenex and Amaral, 2000; Suzuki and Eichenbaum, 2000; Norman and O'Reilly, 2003), that whatever the different roles of individual structures in the medial temporal lobe may be, the division of labor among these structures is not absolute. Further, we suggest that whatever the division of labor may be, it is unlikely to fall along the lines of a simple, logical dichotomy. The operation of this highly interconnected and dynamic system is bound to be more complex and we may not be well served by attempting to understand it using simple dissociations.
